# Hospitals

**Published:** 1849-01

**Authors:** 


### ECLECTIC DEPARTMENT,

### AND SPIRIT OF THE MEDICAL PERIODICAL PRESS.

---

*Hospitals.*—The following historical sketch of the institution of hospitals, and other establishments of charity, is copied from the *Buffalo Daily Commercial Advertiser,* and will, we think, be perused with interest by our readers.—Editor.

*Hospitals—Establishments of Charity*—No ancient legislator ever proposed a hospital for the poor and infirm, or a hospital for the strangers and destitute. When peasants, or any wanderers from the country, came into Rome, if they did not leave it after the market, they had no resource but to pass the night in the Arcades, and about the forum, or in the porches before the temples. The Greeks were ignorant even of the name of an hospital; the word "nosocomium" was first employed by St. Jerome and St. Isidore. It is true in the pyrytaneum at Athens, there was provided subsistence for the wives and children of those who had suffered for their country; but there was no asylum in sickness.

The infirm and sick are wholly overlooked in the institutions of Lycurgus, as in those of all other legislators of Greece, although the Father of Medicine, Hippocrates, with a solemn oath, swears that he will visit all his life the poor gratuously.

In ancient Rome there was the same neglect and indifference in regard to the poor. Numa made no provision for them; and during the Republic the bounties of the state were given only to those who were in health. The Emperors were not more humane, though it is true certain baths, or thermæ, were consecrated to the use of the people. The rich used to give daily to their poor clients the sportula, of which Juvenal speaks so often; but there was no public asylum for the poor, and in sickness they were left to expire beneath their own miserable tiles, which afforded no shelter from sun or rain. The slaves were left unburied; so that Horace speaks of the Esquiline hill as whitened by the number of bones collected by carniverous birds. Cato, whom Plutarch remarks for living familiarly with his slaves as companions in the labors of husbandry, never thought of providing for them in sickness or old age; and in his book of instructions, De Re Rustica, he prescribes, as an important point of domestic economy, to sell off old slaves, in order not to nourish, he says, useless persons. Neither the religion nor the philosophy of Greece and Rome tended to comfort the poor. The divinities were cruel; the Stoic affected to despise the sufferings of the indigent; the Epicurean took no thought of them. In this respect Paganism was every where the same. Throughout the vast regions of Mogul, India, and China, the use of hospitals is unknown to this day. In no country did Christianity find such institutions existing.

In respect to institutions of mercy, all countries which had not beheld the light of faith were equally destitute. It seems unaccountable, therefore, that so grave an historian as Niebuhr, sould seize the occasion, when speaking of building the Latin hospital, in the twelfth century, to denominate that epoch, the midnight of barbarism at Rome. Truly, it was a blessed night which beheld such foundations, even though their walls may have been built with fragments of statues, and other works of Grecian art. The history of their rise and progress can be traced in a few words. In the year 380, the first hospital in the West was founded by Fabiola, a devout Roman lady, without the walls of Rome. St. Jerome says, expressly, that this was the first of all. And he adds, "That it was a country house destined to receive the sick and infirm, who before used to lie stretched on the public way." The Pilgrim's hospital at Rome, built by Cammachius, became also celebrated. In 330, the priest Zotichus, who had followed Constantine to Byzantium, established in that city, under his protection, a hospice for strangers and pilgrims. The house was built on the plan of the hospice at Jerusalem, which Hircan had erected there one hundred and fifty years before Christ, in expiation for having opened and plundered the tomb of David, and in order to convert the riches he had found there to a benevolent purpose; but it is supposed by Monges that this hospital was only open during the feast of the passover. St. Isidore says, in his Etymologies, "That this was the first hospice for strangers." St. Basil, who founded the first hospitals of Asia, mentions a house for the reception of the sick and of travellers, built on a spot formerly uninhabited, near the city of Cesarea, which became afterwards the ornament of the country and like a second city. St. Basil used frequently to visit it, in order to instruct and console the poor, St. Chrysostom built several hospitals at Constantinople. Justinian, in the year 350, erected at Jerusalem the famous hospital of St. John; and his example was followed by his successors with such zeal, that according to Ducange, in his commentary on the Byzantine History, there were thirty-five establishments of charity in that city alone: there was the Nosocomium, or asylum for the sick; the Xenodochium, for pilgrims and strangers; the Ctochium, or hospice for the poor; the Brephotrophium, or house of education for poor children; the Orphanotrophium, or house for orphans; the Gerocomium, or asylum for the aged; the Candochreum, or gratuitous inn; and the Morotrophium, or house for lunatics.

St Augustin says, "that hospitals have their origin in the truth of religion." In a material sense, too, they owed their existence to the ministers of religion; for, in fact, the first hospitals were the bishops' houses. But as the Episcopal resources proved insufficient, the church decreed that the canons should give the tenth of their revenues and oblations, to maintain the sick poor. In early times the hospitals were always under the direction of priests; thus St. Isidore presided over that at Alexandria, in the time of the Patriarch Theophilus. It was determined by Charlemagne, 816, that at each see one of the canons should always govern the hospital, and that this asylum should be everywhere near the cathedral, in order that the clergy might easily visit it. The consequence of this early discipline can be seen at Paris, where the Hotel Dieu is in the place before the cathedral, and at Brussels, where the great hospital adjoins the church of St. John, which is one of the oldest in that city. Lanfranc, and many other great English prelates, are recorded to have signalized the first year of their piscopacy by erecting houses for the reception of the sick.

